# New perspective in diagnostics of mitochondrial disorders: two years’ experience with whole-exome sequencing at a national paediatric centre

**DOI:** 10.1186/s12967-016-0930-9

**Published:** 2016-06-12

**Authors:** Ewa Pronicka, Dorota Piekutowska-Abramczuk, Elżbieta Ciara, Joanna Trubicka, Dariusz Rokicki, Agnieszka Karkucińska-Więckowska, Magdalena Pajdowska, Elżbieta Jurkiewicz, Paulina Halat, Joanna Kosińska, Agnieszka Pollak, Małgorzata Rydzanicz, Piotr Stawinski, Maciej Pronicki, Małgorzata Krajewska-Walasek, Rafał Płoski

**Affiliations:** Department of Medical Genetics, The Children’s Memorial Health Institute, 04-730 Warsaw, Poland; Department of Paediatrics, Nutrition and Metabolic Diseases,, The Children’s Memorial Health Institute, Warsaw, Poland; Department of Pathology, The Children’s Memorial Health Institute, Warsaw, Poland; Department of Biochemistry and Experimental Medicine, The Children’s Memorial Health Institute, Warsaw, Poland; Department of Radiology, The Children’s Memorial Health Institute, Warsaw, Poland; Department of Medical Genetics, Warsaw Medical University, Pawińskiego str, 02-106 Warsaw, Poland; Department of Genetics, Institute of Physiology and Pathology of Hearing, Nadarzyn, Poland

**Keywords:** Whole-exome sequencing, Mitochondrial disorders, Mitochondrial disease criteria scale, Neonates, Basal ganglia involvement, Leigh syndrome, 3-methylglutaconic aciduria, Novel mutation, Candidate gene

## Abstract

**Background:**

Whole-exome sequencing (WES) has led to an exponential increase in identification of causative variants in mitochondrial disorders (MD).

**Methods:**

We performed WES in 113 MD suspected patients from Polish paediatric reference centre, in whom routine testing failed to identify a molecular defect. WES was performed using TruSeqExome enrichment, followed by variant prioritization, validation by Sanger sequencing, and segregation with the disease phenotype in the family.

**Results:**

Likely causative mutations were identified in 67 (59.3 %) patients; these included variants in mtDNA (6 patients) and nDNA: X-linked (9 patients), autosomal dominant (5 patients), and autosomal recessive (47 patients, 11 homozygotes). Novel variants accounted for 50.5 % (50/99) of all detected changes. In 47 patients, changes in 31 MD-related genes (*ACAD9, ADCK3, AIFM1*, *CLPB*, *COX10*, *DLD*, *EARS2*, *FBXL4*, *MTATP6*, *MTFMT, MTND1, MTND3, MTND5*, *NAXE*, *NDUFS6*, *NDUFS7*, *NDUFV1*, *OPA1*, *PARS2*, *PC*, *PDHA1*, *POLG*, *RARS2*, *RRM2B*, *SCO2*, *SERAC1*, *SLC19A3*, *SLC25A12*, *TAZ, TMEM126B, VARS2*) were identified. The *ACAD9*, *CLPB*, *FBXL4*, *PDHA1* genes recurred more than twice suggesting higher general/ethnic prevalence. In 19 cases, variants in 18 non-MD related genes (*ADAR*, *CACNA1A*, *CDKL5*, *CLN3*, *CPS1*, *DMD*, *DYSF*, *GBE1*, *GFAP*, *HSD17B4*, *MECP2*, *MYBPC3*, *PEX5*, *PGAP2, PIGN*, *PRF1*, *SBDS*, *SCN2A*) were found. The percentage of positive WES results rose gradually with increasing probability of MD according to the Mitochondrial Disease Criteria (MDC) scale (from 36 to 90 % for low and high probability, respectively). The percentage of detected MD-related genes compared with non MD-related genes also grew with the increasing MD likelihood (from 20 to 97 %). Molecular diagnosis was established in 30/47 (63.8 %) neonates and in 17/28 (60.7 %) patients with basal ganglia involvement. Mutations in *CLPB*, *SERAC1*, *TAZ* genes were identified in neonates with 3-methylglutaconic aciduria (3-MGA) as a discriminative feature. New MD-related candidate gene (*NDUFB8*) is under verification.

**Conclusions:**

We suggest WES rather than targeted NGS as the method of choice in diagnostics of MD in children, including neonates with 3-MGA aciduria, who died without determination of disease cause and with limited availability of laboratory data. There is a strong correlation between the degree of MD diagnosis by WES and MD likelihood expressed by the MDC scale.

**Electronic supplementary material:**

The online version of this article (doi:10.1186/s12967-016-0930-9) contains supplementary material, which is available to authorized users.

## Background

The diagnostics of mitochondrial disorders (MD) remains a challenge due to clinical heterogeneity [1] and the constantly expanding amount of gene candidates [2] as well as new phenotypes of these conditions [3]. There are eight published studies evaluating diagnostic utility of next generation sequencing (NGS) in mitochondrial patient cohorts, selected either based on particular biochemical signatures of disease [4–8] or centre/cohort-based studies [9–11]. However, of these only four used whole exome sequencing (WES) [7–10].

A particular challenge is the diagnosis of MD in neonates below 3 months of age as these patients may account for up to 30 % of all MD cases [12, 13]. However, so far, this group has not been specifically focused on in terms of diagnostic effectiveness of WES. The prevailing majority (96.5 %) of cases with a molecular diagnosis of MD established at our national reference centre until 2013 included children older than 3 months, indicating considerable under-diagnosis rates in the youngest infants in the Polish population. We have achieved some improvement in neonatal MD detection by performing targeted DNA sequencing (frequently *post mortem*) in cases of neonates with lactic aciduria (LA-uria) found in selective GC–MS screening, including over 90 % of *SCO2* [14] and *DGUOK* [15] deficiencies, and ~ 50 % of *SURF1* deficiency [16].

The purpose of our study was to evaluate WES as a tool for diagnosis of MD depending on the disease probability assessed according to mitochondrial disease criteria (MDC) [17]. We considered both patients with full-range mitochondrial diagnostics (Leigh syndrome features in MRI and/or muscle biopsy evaluation) and those in whom only fragmentary clinical data e.g. abnormal result of GC–MS screening indicating the presence LA-uria and/or 3-methylglutaconic aciduria (3-MGA-uria) were available.

## Methods

### Patients

WES was performed in patients with probable or possible MD, in whom a molecular defect had not been identified within the analysed period. In the retrospective subgroup (88/113 patients) the lag time was 2–25 years (mean 7.5 +/5.9 years). Since 2013 WES has been considered in consecutive patients (25/113). To undergo WES, a patient had to fulfil at least one of the following criteria: 1/neonatal onset; 2/basal ganglia involvement (Leigh syndrome—LS, nonspecific basal ganglia involvement); 3/increased 3-MGA in urine (patients recruited from a group of >250 cases of 3-MGA aciduria identified by national selective GC–MS screening for metabolic disorders since 2000), and 4/genetic counselling demands. Access to biological material and informed consent of parents were *sine qua non* conditions for participation in the study. Details of criteria for patient selection and their clinical characteristics are shown in Table 1 and Additional file 1: Table S1.

**Table 1:** Characteristics of 113 MD suspected patients; inclusion criteria

ID patient	Sex	Date of birth (year)	Neonatal onset	3-MGA in urine	Basal ganglia involvement	Death	MDC score	Muscle biopsy	Period from onset to WES (year)
1	F	2009	+			+	5		5
2	F	2013	+			+	4	Autopsy	0
3	M	2012	+				5	+	2
4	F	2007					4	+	0
5	F	2013	+	+		+	5		0
6	F	2011					4		0
7	M	2006			+		5	+	7
8	M	2008		+			2		0
9	M	2011				+	6	+	2
10	M	2004			+		5	+	7
11	M	2005					2		2
12	M	2005				+	3	Autopsy	7
13	M	2014	+	+		+	4	Autopsy	0
14	F	2006				+	3	+ Autopsy	7
15	F	2008	+	+			4	+	5
16	M	2012			+		3		0
17	F	1992			+		3	+	21
18	F	2003			+		3	+	7
19	M	2009					5	+	3
20	M	2009					4		2
21	F	2006				+	6	+	8
22	M	2010		+	+		8	+	2
23	M	2011	+			+	4	+	3
24	F	2008	+				6	+	4
25	M	2010	+			+	7	+	3
26	M	2011	+		+		8	+	2
27	M	2008	+	+		+	5		6
28	M	2004	+	+		+	3	+	11
29	F	2007	+				5	+	7
30	F	2002	+			+	2	+	13
31	F	2005			+		6	+	9
32	M	2002			+	+	5	Autopsy	3
33	F	2006					3		2
34	M	2006			+		6	+	4
35	M	2012				+	6	+	2
36	M	2006			+	+	5	+	6
37	M	2003	+	+	+		7	+	12
38	M	1985					3	+	12
39	M	1996					3	+	11
40	M	2010	+	+		+	5	Autopsy	4
41	F	2011			+		4	+	3
42	F	2013					2		0
43	M	1967				+	2		10
44	F	1956					4		3
45	F	1995					2	+	11
46	M	2009					3	+	4
47	M	2013					2		0
48	F	2007					2		4
49	M	2012	+			+	6	Autopsy	2
50	M	2009	+			+	2	Autopsy	5
51	M	2003	+		+	+	5	+	12
52	F	2011	+				5		3
53	M	2007					6	+	7
54	M	1990				+	6	+	25
55	F	1981					4	+	21
56	F	2012					4		0
57	M	2010			+		6	+	0
58	M	2012			+		6	+	0
59	F	2010	+				6	+	4
60	M	2003	+		+	+	6	+	10
61	M	1989				+	8	+	23
62	M	1997	+			+	6	+	18
63	F	1989					4	+	16
64	F	2012			+	+	6	+	2
65	M	1991	+		+		4	+	23
66	F	2012	+				5	+	2
67	F	2014	+	+			4		0
68	M	2012			+		4	+	0
69	M	2013					3		0
70	F	2004				+	5	+	11
71	M	2001	+			+	5	+	14
72	M	2011	+			+	4	Autopsy	3
73	F	2002	+				3	+	11
74	F	1989					4	+	12
75	M	2008	+			+	5	+	6
76	F	2003			+		4	+	6
77	F	2011	+		+	+	6	+	3
78	M	1994		+			3		17
79	M	2004					3	+	6
80	F	2012		+		+	2		0
81	F	1990	+			+	4	+ Autopsy	21
82	F	2000			+		3		2
83	F	2003	+			+	4	+	12
84	M	2010	+				3	+	4
85	F	2013			+	+	3		0
86	M	2008				+	2		5
87	M	2010					3		0
88	M	1997					2		0
89	F	2004	+		+	+	4	+	11
90	M	2002				+	4	+	13
91	M	2009				+	6	+	5
92	M	1995		+			2		5
93	M	2011	+			+	3	Autopsy	3
94	F	2010	+	+			4		3
95	F	2011	+				4	+	3
96	M	2011				+	2		2
97	M	2005	+			ND	4	+	10
98	F	2012	+	+		+	2	Autopsy	2
99	F	1974					2		0
100	M	2009				+	3	+	5
101	M	2012			+		4		0
102	F	2006			+		3		0
103	F	2008	+	+		+	3	Autopsy	4
104	F	1988			+		4	+	18
105	F	2014	+			+	5		0
106	M	2011	+				3	+	2
107	M	2006					4	+	8
108	M	2012	+				3	+	2
109	M	1997	+			+	4	Autopsy	18
110	M	2010					2	+	4
111	F	2014	+			+	4		0
112	F	2010	+			+	3	Autopsy	4
113	M	2013				+	4	+	0

*F* female, *M* male

The study included cases with a high probability of MD and those in whom MD was considered possible. The level of probability was assessed according to the MDC score proposed by the Nijmegen mitochondrial team as follows: 2–4 points: MD possible; 5–8 points: MD probable [17]. The MDC scoring for this study did not include the results of muscle biopsy (panels A+B, without C). The mean MD score in the study group was 4.1 ± 1.5 (range 2–8). Muscle biopsy with subsequent OXPHOS evaluation was performed in 67 cases, and autopsy in 15 cases. The family history was positive in 26 cases and three couples were consanguineous.

In the retrospective group, DNA was isolated from fibroblast cultures or frozen tissue samples obtained by muscle/liver biopsy or by autopsy. Whenever possible, skeletal muscle was preferred. In the remaining cases, DNA was isolated from blood. Throughout the paper the genes were classified as MD-related if they had a connection with mitochondrial disorders documented in the literature [9] or non MD-related when this was not the case.

Parents of the patients gave informed consent for the WES analysis. The study protocol was in agreement with the Helsinki Convention and the study was approved by the Ethics Committee of The Children’s Memorial Health Institute.

### Whole-exome sequencing

WES was performed using TruSeqExome Enrichment Kits according to the manufacturer’s instructions (Illumina). The samples were run on 1/4 of a lane on HiSeq 1500 using 2 × 100 bp paired-end reads. Bioinformatics analysis was performed as previously described [18]. Briefly, after initial processing with CASAVA, the sequencing reads were aligned to the hg19 reference genome with the Burrows-Wheeler Alignment Tool and further processed by Genome Analysis Toolkit [19]. Base quality score recalibration, indel realignment, duplicate removal, and SNP/INDEL calling were done as described [20]. The detected variants were annotated using Annovar and converted to MS Access format for final manual analyses. Alignments were viewed with Integrative Genomics Viewer [21, 22]. The complete results of WES, including VCF and/or FASTQ files, are available on demand to qualified researchers. All samples were sequenced so that min. 80 % of target was covered 20× or more.

The presence of the variants identified by WES was confirmed by Sanger sequencing.

## Results

Among 67 probands, we found 99 variants in 49 different genes with a Known disease link (Table 2). They were variants in mtDNA (6 patients) and nuclear DNA (nDNA): X-linked (9 patients), autosomal dominant (5 patients), and autosomal recessive (47 patients), including 11 homozygotes. In 50.5 % (50/99) the detected variants were novel (Table 3). Sixty-six of the variants found in the study group occurred in MD-related genes, whereas 31 were found in non MD-related loci. In addition, deleterious variants in a gene not previously linked to disease in humans were identified in one proband (Table 2).

**Table 2:** Molecular variants identified in 67 individuals of the study group

Gene	Chromosome:RefSeq	Variant 1			Variant 2			Zygosity status	Mode	ID patient
		Type	Status	Origin	Type	Status	Origin			
Mitochondrial disease gene										
*ACAD9*	chr3:NM_014049.4	c.514G>A/p.Gly172Arg	Novel	mat	c.803C>T/p.Ser268Phe	Novel	pat	comphtz	AR	15
*ACAD9*	chr3:NM_014049.4	c.1552C>T/p.Arg518Cys	Known	mat	c.1553G>A/p.Arg518His	Known	pat	comphtz	AR	23
*ACAD9*	chr3:NM_014049.4	c.728C>G/p.Thr243Arg	Novel	ND	c.1552C>T/p.Arg518Cys	Known	mat	comphtz	AR	53
*ADCK3*	chr1:NM_020247.4	c.827A>G/p.Lys276Arg	Novel	mat	c.1702delG/p.Gly568Argfs	Novel	pat	comp htz	AR	61
*AIFM1*	chrX:NM_004208.3	c.1474T>C/p.Tyr492His	Novel	mat	–		–	hemi	XLR	25
*CLPB*	chr11:NM_030813.4	c.2045T>A/p.Ile682Asn	Known	pat	c.1937_1938insG/p.645Gly_646Cysfs	Known	mat	comphtz	AR	5
*CLPB*	chr11:NM_030813.4	c.1249C>T/p.Arg417^a^	Known	pat	c.748C>T/p.Arg250^a^	Known	mat	comphtz	AR	27
*CLPB*	chr11:NM_030813.4	c.1249C>T/p.Arg417^a^	Known	pat	c.1222A>G/p.Arg408Gly	Known	mat	comphtz	AR	67
*COX10*	chr17:NM_001303.3	c.1030A>G/p.Met344Val	Novel	pat	c.1270dupC/p.Leu424Profs	Novel	mat	comphtz	AR	9
*COX10*	chr17:NM_001303	c.674C>T/p.Pro225Leu	Known	mat	c.674C>T/p.Pro225Leu	Known	pat	hom	AR	36
*DLD*	chr7:NM_000108.4	c.1123G>A/p.Glu375Lys	Known	mat	c.1123G>A/p.Glu375Lys	Known	pat	hom	AR	31
*EARS2*	chr16:NM_001083614.1	c.164G>A/p.Arg55His	Known	mat	c.325G>C/p.Gly109Arg	Novel	pat	comphtz	AR	7
*EARS2*	chr16:NM_001083614.1	c.164G>A/p.Arg55His	Known	pat	c.1256C>T/p.Pro419Leu	Novel	mat	comphtz	AR	70
*FBXL4*	chr6:NM_012160.4	c.858+1G>T/p.?	Novel	pat	c.585+5G>C/p.?	Novel	mat	comphtz	AR	3
*FBXL4*	chr6:NM_012160.4	c.1303C>T/p.Arg435^a^	Known	ND	c.64C>T/p.Arg22^a^	Novel	mat	comphtz	AR	52
*FBXL4*	chr6:NM_012160.4	c.64C>T/p.Arg22^a^	Novel	mat	c.64C>T/p.Arg22^a^	Novel	pat	hom	AR	55
*MTATP6*	chrM:NC_012920.1	m.9185T>C/p.Leo220Pro	Known	mat	–		–	hompl	M	32
*MTFMT*	chr15:NM_139242.3	c.994C>T/p.Arg332^a^	Known	ND	c.626C>T/p.Ser209Leu	Known	ND	comphtz	AR	91
*MTND1*	chrM:NC_012920.1	m.3902_3908invACCTTGC/p.?	Known	de novo	–		–	hetpl	M	22
*MTND1*	chrM:NC_012920.1	m.3688G>A/p.Ala128Thr	Known	ND	–		–	hompl	M	64
*MTND3*	chrM:NC_012920.1	m.10254G>A/p.Asp66Asn	Known	de novo	–		–	hetpl	M	57
*MTND5*	chrM:NC_012920.1	m.12706T>C/p.Phe124Leu	Known	de novo	–		–	hetpl	M	34
*MTND5*	chrM:NC_012920.1	m.13513G>A/p.Asp393Asn	Known	de novo	–		–	hetpl	M	35
*NAXE*	chr1:NM_144772.2	c.653A>T/p.Asp218Val	Known	mat	c.743_744delC/p.247Ala_248Thrfs	Known	pat	comphtz	AR	12
*NDUFS6*	chr5:NM_004553.4	c.313_315delAAAG/p.104Lys_106Thrfs	Novel	pat	c.334_359del26ins13/p.Glu112 fs	Novel	mat	comphtz	AR	1
*NDUFS7*	chr19:NM_024407.4	c.376C>T/p.Leu126Phe	Novel	ND	c.504G>C/p.Arg168Ser	Novel	ND	het	AR	75
*NDUFV1*	chr11:NM_007103.3	c.733G>A/p.Val245Met	Novel	pat	c.383G>T/p.Arg128Leu	Novel	mat	comphtz	AR	10
*OPA1*	chr3:NM_015560.2	c.1146A>G/p.Ile382Met	Known	mat	–		–	htz	AD	33
*PARS2*	chr1:NM_152268.3	c.1091C>G/p.Pro364Arg	Novel	mat	c.239T>C/p.Ile80Thr	Novel	pat	comphtz	AR	60
*PC*	chr11:NM_000920.3	c.808C>T/p.Arg270Trp	Known	pat	c.2381_2383delTGG/p.Val794del	Novel	mat	comphtz	AR	29
*PC*	chr11:NM_000920.3	c.1487G>A/p.Arg496Gln	Novel	ND	c.584C>T/p.Ala195Val	Novel	ND	comphtz	AR	71
*PDHA1*	chr X:NM_000284.3	c.262C>T/p.Arg88Cys	Known	mat	–		–	hemi	XLD	19
*PDHA1*	chrX:NM_000284.3	c.856_859dupACTT/p. Arg288Leufs	Novel	de novo	–		–	htz	XLD	56
*PDHA1*	chrX:NM_000284.3	c.933_935del/p.Arg311del l	Known	de novo	–		–	htz	XLD	66
*PDHA1*	chrX:NM_000284.3	c.291G>A/p.?	Novel	de novo	–		–	hemi, mosaic	XLD	68
*POLG*	chr15:NM_001126131.1	c.2639C>A/p.Ala880Asp	Novel	pat	c.2243G>C/p.Trp748Ser	Known	mat	comphtz	AR	113
*RARS2*	chr6:NM_020320.3	c.1026G>A/p.Met342Ile	Novel	mat	c.622C>T/p.Gln208^a^	Novel	pat	comphtz	AR	41
*RRM2B*	chr8:NM_015713.4	c.414_415delCA/p.Tyr138^a^	Novel	mat	c.414_415delCA/p.Tyr138^a^	Novel	ND	hom	AR	21
*RRM2B*	chr8:NM_015713.4	c.686G>T/p.Gly229Val	Known	mat	c.686G>T/p.Gly229Val	Known	pat	hom	AR	51
* SCO2*	chr22:NM_005138.2	c.418G>A/p.Glu140Lys	Known	ND	c.418G>A/p.Glu140Lys	Known	ND	hom	AR	54
*SERAC1*	chr6:NM_032861.3	c.1822_1828+10delinsACCAACAGG	Known	ND	c.1822_1828+10delinsACCAACAGG	Known	ND	hom	AR	37
*SLC19A3*	chr2:NM_025243.3	c.68G>T/p.Gly23Val	Known	Pending	c.68G>T/p.Gly23Val	Known	Pending	hom	AR	58
*SLC19A3*	chr2:NM_025243.3	c.74dupT/p.Ser26Leufs	Known	ND	c.74dupT/p.Ser26Leufs	Known	ND	hom	AR	109
*SLC25A12*	chr2:NM_003705.4	c.1335C>A/p.Asn445Lys	Novel	mat	c.1335C>A/p.Asn445Lys	Novel	pat	hom	AR	24
*TAZ*	chrX:NM_000116.3	c.684_685insC/p.227Phe_228Profs	Novel	ND	–		–	hemi	XLR	28
*TMEM126B* ^a^	chr11:NM_018480.4	c.635G>T/p.Gly212Val	Known	mat	c.635G>T/p.Gly212Val	Known	pat	hom	AR	59
*VARS2*	chr6:NM_001167734.1.5	c.1100C>T/p.Thr367Ile	Known	Pending	c.1490G>A/p.Arg497His	Novel	Pending	comphtz	AR	97
Non mitochondrial disease gene										
*ADAR*	chr1:NM_001111.4	c.3202+1G>A/p.?	Novel	ND	c.577C>G/p.Pro193Ala	Known	ND	comphtz	AR	18
*CACNA1A*	chr19:NM_001127221.1	c.1997C>T/p.Thr666Met	Known	mat	–		–	htz	AD	39
*CDKL5*	chrX:NM_003159.2	c.1942C>T/p.Gln648^a^	Novel	mat	–		–	hemi	XLD	65
*CLN3*	chr16:NM_001042432.1	c.954_962+18del27/p.Leu313_Trp321del	Known	pat	c.461-280_677+382del966	Known	Pending	comphtz	AR	88
*CPS1*	chr2:NM_001875.4	c.1837-8A>G/p.?	Known	mat	c.3691G>C/p.Ala1231Pro	Novel	Paternal	comphtz	AR	13
*CPS1*	chr2:NM_001875.4	c.1289C>G/p.Ser430^a^	Novel	mat	c.3971_3972delT/p.1323Ile_1324Leufs	Novel	pat	comphtz	AR	40
*DMD*	chr X:NM_004006	c.31+1G>A/p.?	Novel	mat	–		–	hemi	XLR	38
*DYSF*	chr2:NM_003494.3	c.1180+5G>A/p.?	Known	ND	c.6124C>T/p.Arg2042Cys	Known	ND	comphtz	AR	45
*GBE1*	chr3:NM_000158.3	c.1621A>T/p.Asn541Tyr	Novel	mat	c.263G>A/p.Cys88Tyr	Novel	pat	comphtz	AR	14
* GFAP*	chr17:NM_002055.4	c.1100G>C/p.Arg367Thr	Novel	de novo	–		–	htz	AD	42
*HSD17B4*	chr5:NM_000414.3	c.46G>A/p.Gly16Ser	Known	ND	c.367C>T/p.His123Tyr	Novel	ND	comphtz	AR	30
*MECP2*	chrX:NM_004992.3	c.89delA/p.Lys30Argfs	Novel	de novo	–		–	hemi	XLD	106
*MYBPC3*	chr11:NM_000256.3	c.1351+1G>A/p.?	Known	pat	–		–	htz	AD	8
*PEX5*	chr12:NM_001131025.1	c.1669C>T/p.Arg557Trp	Known	mat	c.1799C>T/p.Ser600Leu	Novel	pat	comphtz	AR	20
*PGAP2*	chr11:NM_001256240.1	c.2T>G/p.Met1?	Known	mat	c.221G>A/p.Arg74His	Known	pat	comphtz	AR	73
*PIGN*	chr18:NM_176787.4	c.932T>G/p.Leu311Trp	Known	mat	c.790G>A/p.Gly264Arg	Known	pat	comphtz	AR	6
*PRF1*	chr10:NM_001083116.1	c.808_812delGGCAG/p.Gly270 fs	Novel	mat	c.658G>A/p.Gly220Ser	Known	pat	comphtz	AR	2
*SBDS*	chr7:NM_016038.2	c.258+2T>C/p.?	Known	pat	c.184A>T/p.Lys62^a^	Novel	mat	comphtz	AR	95
*SCN2A*	chr2:NM_021007.2	c.2948T>G/p.Leu983Trp	Novel	de novo	–		–	htz	AD	47
New candidate gene for mitochondrial disease										
*NDUFB8*	chr10:NM_005004.3	c.432C>G/p.Cys144Trp	Novel	mat	c.227C>A/p.Pro76Gln	Novel	pat	comphtz	AR	26

*mat* maternal, *pat* paternal, *ND* not determined (DNA not available), *hom* homozygote, *htz* heterozygote, *comp htz* compound heterozygote, *hemi* hemizygote, *hompl* homoplasmic, *hetpl* heteroplasmic, *AR* autosomal recessive inheritance, *AD* autosomal dominant inheritance, *XLR* X-linked recessive inheritance, *XLD* X-linked dominant inheritance, *M* mitochondrial inheritance

^a^Data published on ESHG 2016 by Alston et al.

**Table 3:** Novel molecular variants identified in the study; pathogenicity status

Gene	Variant	MAF		Pathogenicity status^a^	Genotype–Phenotype correlation^b^	Parental results status	Family history	ID patient
		1000 G	POL 400					
*ACAD9*	c.514G>A/p.Gly172Arg	0	0	Pathogenic	Moderate	in-trans	Negative	15
*ACAD9*	c.803C>T/p.Ser268Phe	0	0	Pathogenic	Moderate	in-trans	Negative	15
*ACAD9*	c.728C>G/p.Thr243Arg	0	0	Pathogenic	Low	in-trans	Negative	53
*ADAR*	c.3202+1G>A/p.?	0	0.0014	Pathogenic	Moderate	ND	Affected brother	18
*ADCK3*	c.827A>G/p.Lys276Arg	0	0	Pathogenic	High	in-trans	Negative	61
*ADCK3*	c.1702delG/p.Gly568Argfs	0	0	Pathogenic	High	in-trans	Negative	61
*AIFM1*	c.1474T>C/p.Tyr492His	0	0	Pathogenic	Moderate	X-linked	Negative	25
*CDKL5*	c.1942C>T/p.Gln648^a^	0	0	Pathogenic	Moderate	X-linked	Negative	65
*COX10*	c.1030A>G/p.Met344Val	0	0.0007	Pathogenic	Moderate	in-trans	Negative	9
*COX10*	c.1270dupC/p.Leu424Profs	0	0	Pathogenic	Moderate	in-trans	Negative	9
*CPS1*	c.3691G>C/p.Ala1231Pro	0	0.0014	Pathogenic	Low	In-trans	Affected sister	13
*CPS1*	c.1289C>G/p.Ser430^a^	0	0.0014	Pathogenic	Moderate	in-trans	Affected brother	40
*CPS1*	c.3971_3972delT/p.1323Ile_1324Leufs	0	0.0014	Pathogenic	Moderate	in-trans	Affected brother	40
*DMD*	c.31+1G>A/p.?	0	0	Pathogenic	Low	X-linked	Affected many males	38
*EARS2*	c.325G>C/p.Gly109Arg	0	0.0014	Likely pathogenic	High	in-trans	Negative	7
*EARS2*	c.1256C>T/p.Pro419Leu	0	0	Likely pathogenic	Moderate	in-trans	Negative	70
*FBXL4*	c.858+1G>T/p.?	0	0	Pathogenic	High	in-trans	Miscarriage	3
*FBXL4*	c.585+5G>C/p.?	0	0	Pathogenic	High	in-trans	Miscarriage	3
*FBXL4*	c.64C>T/p.Arg22^a^	0	0	Pathogenic	Moderate	in-trans	Empty ovum	52
*FBXL4*	c.64C>T/p.Arg22^a^	0	0	Pathogenic	Moderate	in-trans	Negative	55
*GBE1*	c.1621A>T/p.Asn541Tyr	0	0	Pathogenic	Moderate	in-trans	Negative	14
*GBE1*	c.263G>A/p.Cys88Tyr	0	0	Possibly pathogenic	Moderate	in-trans	Negative	14
*GFAP*	c.1100G>C/p.Arg367Thr	0	0	Pathogenic	Moderate	de novo	Negative	42
*HSD17B4*	c.367C>T/p.His123Tyr	0	0.0014	Pathogenic	Moderate	ND	Affected brother	30
*MECP2*	c.89delA/p.Lys30Argfs	0	0.0	Pathogenic	High	de novo	Negative	106
*NDUFB8*	c.432C>G/p.Cys144Trp	0	0.0014	Possibly pathogenic	Moderate	in-trans	Negative	26
*NDUFB8*	c.227C>A/p.Pro76Gln	0	0	Pathogenic	Moderate	in-trans	Negative	26
*NDUFS6*	c.313_315delAAAG/p.104Lys_106Thrfs	0	0	Pathogenic	Moderate	in-trans	Affected brother	1
*NDUFS6*	c.334_359del26ins13/p.Glu112 fs	0	0	Pathogenic	Moderate	in-trans	Affected brother	1
*NDUFS7*	c.376C>T/p.Leu126Phe	0	0	Pathogenic	Moderate	ND	Similar symptoms in brother	75
*NDUFS7*	c.504G>C/p.Arg168Ser	0	0	Likely Pathogenic	Moderate	ND	Similar symptoms in brother	75
*NDUFV1*	c.733G>A/p.Val245Met	0.0005	0	Pathogenic	High	in-trans	Negative	10
*NDUFV1*	c.383G>T/p.Arg128Leu	0	0	Pathogenic	High	in-trans	Negative	10
*PARS2*	c.1091C>G/p.Pro364Arg	0.0014	0.003	Pathogenic	Moderate	in trans	Affected sibs	60
*PARS2*	c.239T>C/p.Ile80Thr	0	0	Pathogenic	Moderate	in trans	Affected sibs	60
*PC*	c.2381_2383delTGG/p.Val794del	0	0	uncertain Pathogenic	High	in-trans	Affected brother	29
*PC*	c.1487G>A/p.Arg496Gln	0	0	Pathogenic	High	ND	Negative	71
*PC*	c.584C>T/p.Ala195Val	0	0	Pathogenic	High	ND	Negative	71
*PDHA1*	c.856_859dupACTT/p. Arg288Leufs	0	0	Pathogenic	High	de novo	Negative	56
*PDHA1*	c.291G>A/p.?	0	0.0000	Uncertain pathogenic	Moderate	de novo	Negative	68
*PEX5*	c.1799C>T/p.Ser600Leu	0	0	Pathogenic	Low	in-trans	Negative	20
*POLG*	c.2639C>A/p.Ala880Asp	0	0	Pathogenic	Moderate	in-trans	Negative	113
*PRF1*	c.808_812delGGCAG/p.Gly270 fs	0	0.0000	Pathogenic	Low	in trans	Negative	2
*RARS2*	c.1026G>A/p.Met342Ile	0	0	Likely pathogenic	Moderate	in-trans	Affected brother	41
*RARS2*	c.622C>T/p.Gln208^a^	0	0.0014	Pathogenic	Moderate	in-trans	Affected brother	41
*RRM2B*	c.414_415delCA/p.Tyr138^a^	0	0.0014	Pathogenic	High	ND	Negative	21
*SBDS*	c.184A>T/p.Lys62^a^	0	0.002	Pathogenic	Low	in-trans	PI neural tube defect	95
*SCN2A*	c.2948T>G/p.Leu983Trp	0	0.0013	Pathogenic	High	de novo	Negative	47
*SLC25A12*	c.1335C>A/p.Asn445Lys	0	0	Pathogenic	Moderate	in-trans	Negative	24
*TAZ*	c.684_685insC/p.227Phe_228Profs	0	0.0012	Pathogenic	Low	ND	Negative	28
*VARS2*	c.1490G>A/p.Arg497His	0	0	Pathogenic	Low	ND	Similar disease in sibs	97

*ND* not determined due to lack of clinical data or DNA not available

^a^Pathogenicity status evaluated according to in silico prediction algorithms (CADD, MetaSVM, Polyphen2 HDIV, Polyphen HVAR, mutation assessor, LRT, MetaLR, SIFT, mutationtaster) and classified as: pathogenic—nonsense, frameshift, splicesite and missense variants with pathogenic status at least in 7 of used algorithms; likely pathogenic - missense variants with pathogenic status in 4–6 of used algorithms; possibly pathogenic—missense variants with pathogenic status <4 of used algorithms

^b^Genotype-Phenotypecorrelationassessed by two independent specialists in clinical genetics and metabolic medicine

Mutations in MD-related genes were found in 47 probands. Identified pathogenic variants in 31 different genes included 27 located in nDNA and 4 in mtDNA (Table 2). Eleven genes were found defective more than once (*PDHA1*-4x, *ACAD9*, *CLPB,* and *FBXL4-*3x, *COX10*, *EARS2*, *MTND1*, *MTND5*, *PC*, *RRM2B*, *SLC19A3-*2x). The majority of these genes were not previously screened for in our mitochondrial diagnostic centre, with the exceptions of *TAZ*, *PDHA1* [23], *SCO2*, and the genes encoding MTND and MTATP subunits. Below we present the results that were analysed according to selected phenotypic features (neonatal onset, basal ganglia involvement, 3-MGA) and MD likelihood.

### Subgroup of neonates

**Fig. 1:**
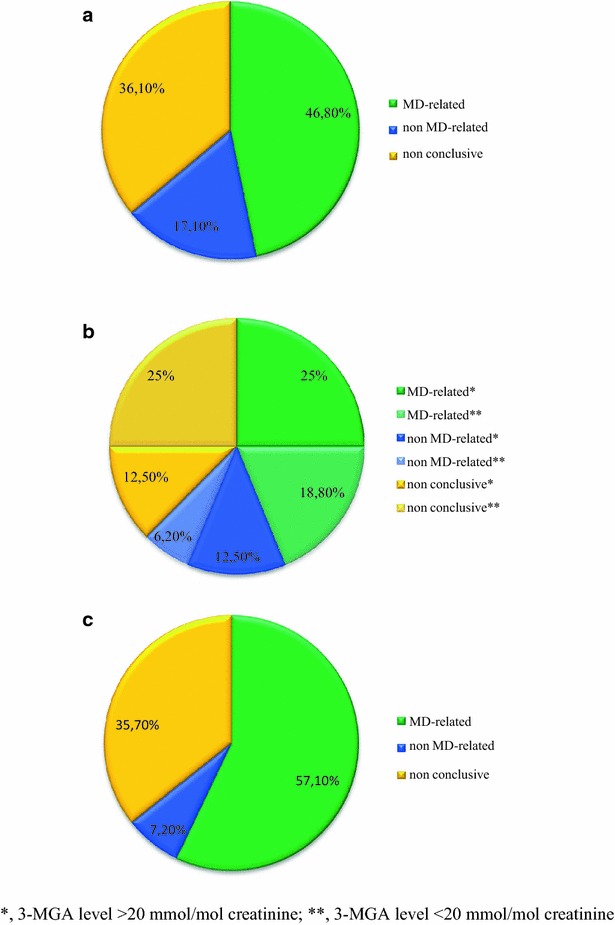
The percentage of detected MD-related genes, non MD-related genes and non-conclusive WES results in (**a**) neonates (n = 47), **b** patients with 3-MGA-uria (n = 16) and **c** patients with basal ganglia involvement (n = 28)

WES yielded conclusive results in 63.9 % (30/47) of neonates studied (Fig. 1a). We found mutations in 23 different genes, including 16 MD-related (*ACAD9*, *AIFM1*, *CLPB*, *FBXL4*, *NDUFS6, NDUFS7, PARS2, PC*, *PDHA1* [23], *RRM2B*, *SERAC1*, *SLC19A3, SLC25A12*, *TAZ, TMEM126B, VARS2*) and 7 non MD-related (*CDKL5, CPS1, HSD17B4, MECP2, PGAP2, PRF1, SBDS*). The majority of the neonates with positive WES results came from the first pregnancy of healthy unrelated parents. Twenty-nine neonates died before establishing a diagnosis; half in the early neonatal period. In 28 cases the mitochondrial testing was completed, including MR imaging and spectroscopy, muscle biopsy and fibroblast culture collection. In the remaining cases, mitochondrial diagnostics were absent or limited only to selective GC–MS screening showing increased excretion of lactate, Krebs cycle metabolites, 3-MGA and/or ketone bodies.

### Subgroup with 3-methylglutaonic aciduria

Positive WES results were obtained in seven of 16 patients with persisting 3-MGA (Fig. 1b). In two subjects [P28 and P37] we found mutations in *TAZ* and *SERAC1* genes known to cause mitochondrial diseases with 3-MGA as a discriminative feature [24]. *Ex post* it was apparent that earlier some important clinical features, including hearing impairment in the patient with *SERAC1* mutations and increased excretion of 3-MGA in the terminal stage in the boy with the *TAZ* mutation, had been overlooked.

In three unrelated 3-MGA neonates included in this study, we identified mutations in the *CLPB* gene, whose link to human disease was subsequently established [25]. Two of them [P5 and P27] have already been reported in the first disease description [25].

Additionally, in two 3-MGA patients [P13, P40] we found molecular variants in the *CPS1*, a non MD-related gene linked to urea cycle disorder. In remaining patients in whom the reason for inclusion in the study group was a single GC–MS assessment (*ACAD9* and *MYBPC3* patients [P15, P8]), increased excretion of 3-MGA has been apparently transient or it was within normal limits after quantitative verification (Additional file 1: Table S1). Since traces of 3-MGA excretion were also found in a number of healthy siblings and parents of the patients the transient or mild increase in patients was most likely without a causal relationship.

### Basal ganglia involvement (Leigh syndrome, Leigh-like, others)

In 15 of 28 patients from this group (Fig. 1c), molecular variants in LS-associated genes, including genes responsible for deficiency of complex I (*MTND1*, *MTND3*, *MTND5*, *NDUFV1*), complex IV (*COX10*), complex V (*MTATP6*), combined OXPHOS defect (*EARS2*, *PARS2, RARS2, RRM2B, SERAC1*, *SLC19A3*), and pyruvate dehydrogenase complex deficiency (*DLD, PDHA1*) [23] were identified. In the remaining 13 patients with LS or other basal ganglia involvement WES did not reveal variants in MD-related genes as listed by Neveling [9].

In three patients with basal ganglia involvement one MD-related candidate *(NDUFB8)* and two known non MD-related genes (*ADAR, CDKL5*) were identified.

### Defects in non MD-related genes

In 19 patients who were included in the study because of a possible (low probability) mitochondrial disease, mutations in various non MD-related genes (*ADAR*, *CACNA1A*, *CDKL5*, *CLN3*, *CPS1*, *DMD*, *DYSF*, *GBE1*, *GFAP*, *HSD17B4*, *MECP2*, *MYBPC3*, *PEX5*, *PGAP2, PIGN*, *PRF1*, *SBDS, SCN2A*) were identified (Table 2; Additional file 1: Table S1).

### New MD-related disorders

While our project was ongoing new candidate genes found by us including *PARS2* [26] and *CLPB* have been described by other research teams [25]. The causal role of another two of our candidates has been recognized even more recently. The *NAXE* gene (*APOA1BP* according to old nomenclature), a susceptibility locus for migraine [27], in which likely pathogenic variants were found by us in two brothers with a fatal encephalitis-like disorder [P12], has been described in April 2016 as the cause of lethal infantile leukoencephalopathy in a large consanguineous family [28]. A homozygous variant in the *TMEM126B* gene encoding a subunit required for mitochondrial complex I assembly [29, 30], found by us in a complex I deficient girl with extra-neurological presentation [P59], has been discovered and verified functionally as a cause of the disease in a subset of other patients (ESHG 2016, Alston et al.).

The interesting remaining candidate for a novel disease gene identified in our study is *NDUFB8*. Compound heterozygosity for two variants in *NDUFB8* was found in a boy with a typical course of LS and complex I deficiency in muscle homogenate [P26] (Additional file 1: Table S1). *NDUFB8* [31] encodes a known subunit of complex I, but, to the extent of our knowledge, its association with complex I deficiency and LS in humans has not been published so far.

### Mitochondrial disease criteria score

In the studied cohort there were 40 patients with high probability of MD, i.e., with an MDC score above 4 (5–8, criteria A+B, without C). Positive WES results were obtained in 36 of them (90 %). In this group, pathogenic variants were found mainly in MD-related genes (*CPS1* being the exception). WES failed in four patients [P49, P62, P77, P105] with an MDC score above 4. Some of them were found to carry a deleterious variant in one of the known MD-related genes only on one allele. The definite diagnosis still remains open in these cases. Bioinformatics tools for identification of structural variants using NGS have not been applied to our data so it is possible that in some cases the disease may be caused by large deletions/duplications. The complete lists of variants detected in the subjects without fully conclusive results and/or the respective FASTQ files are available on demand to qualified researchers.

Intermediate probability of MD (MDC = 4) was associated with the occurrence of variants in both MD-related and non MD-related genes, in ten (10/31) and six (6/31) patients, respectively. MD-related genes were represented in this subset twice by *ACAD9* [P15, P23] and *PDHA1* [P56, P68], and in single cases by *CLPB* [P67], *FBLX4* [P55], *POLG* [P113], *RARS2* [P41], *SLC19A3* [P109], and V*ARS2* [P97].

In the subgroup with low probability of MD, i.e., a MDC score of 2–3 points, positive WES results were obtained in 15 of 42 cases (36 %). Three MD-related genes (7 %) including: *OPA1* [P33], *TAZ* [P28] and *NAXE* [P12] were found. Non MD-related genes were identified in 12 of 42 cases (29 %).

**Fig. 2:**
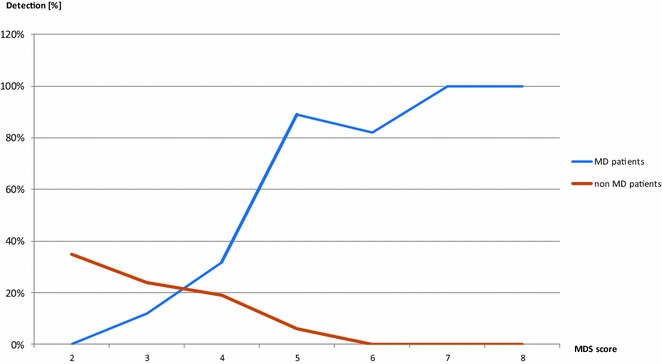
Efficacy of WES in 113 patients with possible or probable mitochondrial pathology depending on the level of probability expressed by MDC Nijmegen score

The percentage of positive results rose gradually as the likelihood of MD increased, as shown by the MDC score (Fig. 2). In the subset of high probability of MD (MDC above 4), the detection percentage reached 90 %. There was a broad range of MD-related genes (Table 2). Only one non MD-related gene (*CPS1*) was found in a neonate with a MDC score of 5.

The participation of detected MD-related genes as compared with non MD-related genes also grew as the likelihood of MD probability increased (from 20 to 97 %, data not shown).

### WES diagnostics of current cases vs. archival DNA samples

Characteristics of the patients stratified by the waiting period between disease onset and WES qualification into archival material and current diagnostics subset is shown in Table 4. WES efficacy assessed as percentage of molecularly confirmed diagnoses was comparable being higher than 50 % in both subsets. Contribution of MD-related genes expressed by the ratio of MD-related/non MD-related genes was higher in the archival than current subset (3.4 vs. 1.0, respectively) indicating that this subset contained more patients with non-mitochondrial genetic disorders and that our current qualification for WES became less demanding.

**Table 4:** WES results related to the origin of the qualified material and to the specific inclusion criteria

Subgroups of patients	MD or non-MD genes loci of variants	Diagnostics based on archival material	Current diagnostics	Total
Disease onset (year)		1996–2012	2013–2014	1996–2014
Number of patients		88 (5.5/year)	25 (12.5/year)	113
Period from onset to WES qualification (years)		2–25 (mean 5.5 ± 5.9 )	0	0–25
MDC scale (A+B, without C)		4.2 ± 1.5 (2–8)	3.6 ± 1.2 (2–6)	4.1 ± 1.5
Ratio of MD-related/non MD related genes		3.4	1.0	2.4
Patients deceased	Total no.	41	8	44 %
	*MD*	*51.2 % (21)*	*2*	*47 % (23)*
	*non MD*	*(3)*	*2*	*(5)*
Patients with neonatal onset	Total no.	41	6	42 %
	*MD*	*53.7 % (22)*	*2*	*51 % (24)*
	*non MD*	*(5)*	*2*	*(7)*
Patients with LS or other basal ganglia involvement	Total no.	21	7	25 %
	*MD*	*61.9 % (13)*	*3*	*57 % (16)*
	*non MD*	*(2)*	*0*	*(2)*
3-methylglutaconic aciduria	Total no.	13	3	14 %
	*MD*	*53.8 % (7)*	*2*	*53 % (9)*
	*non MD*	*0*	*1*	*(1)*
Muscle biopsy	Total no.	62	5	67/113
	*MD*	*56.4 % (35)*	*(4)*	*58 % (39)*
	*non MD*	*(10)*	*(0)*	*(10)*
Percentage of muscle biopsy		70 %	20 %	59 %

^a^Italics in brackets indicates the number of patients in the given subset

*LS* Leigh syndrome, *MD* mitochondrial disorder, *MD/non MD* MD-related/non MD-related genes wherein variants were identified

### Muscle biopsy findings

OXPHOS assessment available for 67 muscle homogenates showed isolated complex I deficiency in 16 cases, complex IV deficiency in 6 cases and combined OXPHOS defect in 10 cases. There were unspecific changes in 22 bioptates and normal OXPHOS activity in 10. The results were not conclusive in three cases due to technical problems (too small muscle specimen, low protein concentration, low citric synthase activity).

Complex I deficiency was found in 11 patients with molecular variants in MD-related genes (*ACAD9* [P15, P23, P53], *NDUFV1* [P10], *NDUFS7* [P75], *MTND1* [P64], *MTND3* [P57], *EARS2* [P7], *SLC19A3* [P58], *TMEM126B* [P59]) and in one candidate (*NDUFB8* [P26]. In one patient [P95] a defect in non MD-related gene (*SBDS)* was found. In 4 patients WES results were not conclusive.

In the subset with complex IV deficiency molecular defects were confirmed in three patients including *COX10* [P9, P36] and *EARS2* [P70]) while three WES analyses were not conclusive.

Combined OXPHOS defect occurred in 8 patients with variants identified in MD-related genes (*FBXL4* [P3], *ADCK3* [P61], *RRM2B* [P21, P51], *AIFM1* [P25], *TAZ* [P28], *PC* [P71], *MTND5* [P34]). In two cases WES results were not conclusive.

Histological and histochemical data of the patients with positive WES showed presence of ragged red fibers in four cases (*ADCK3* [P61], *ACAD9* [P15, P23, P53]), “lipid storage myopathy” in four (*PC* [P71, P29], *MTND5* [P35], *PDHA1* [P66]) and SMA-like pattern in three (*AIFM1* [P25], *SCO2* [P54], *RRM2B* [P51]).

Depletion of mitochondrial DNA (<30 % of reference value) was revealed in tissues of 8 patients. Molecular defect was established by WES in four of them (*COX10* [P9], *FBXL4* [3], *RRM2B* [P21, P51]).

### Verification of mitochondrial genome variants

**Fig. 3:**
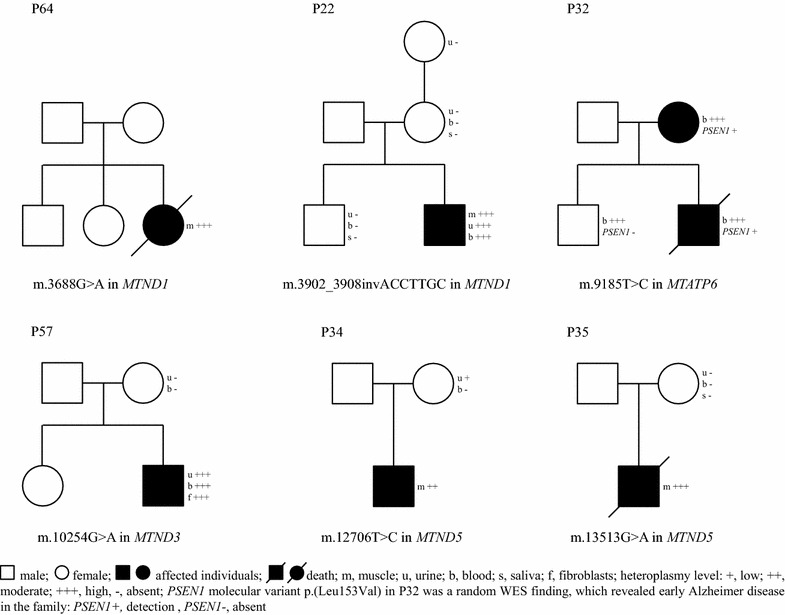
Family study in six probands with mtDNA known mutations

Interestingly, in six patients with typical MD phenotype the search for pathogenic variants in MD-related nuclear genes by WES was negative yet pathogenic variants were found in mtDNA. Each mtDNA variant identified by WES, was subsequently verified by Sanger sequencing using specific primers for mitochondrial genome. All detected changes are known and have been repeatedly reported. Examination of different tissues in probands and maternally related family members showed varying levels of heteroplasmy (Fig. 3).

## Discussion

Our results confirm that the implementation of WES led to a significant breakthrough in the diagnostics of MD in children [32]. This is expressed by both the increased number of identified genes and faster establishment of final diagnosis. The total number of genes with likely causative defects found in the present work was 47, a very satisfactory diagnostic yield when compared with 8 genes identified by us by single-gene Sanger sequencing before the introduction of WES (203 such diagnoses per ~1200 patients studied in the period from 1996 to 2013).

In our study we observed a pronounced upward trend in the detection of the molecular background of mitochondrial diseases that was associated with increased MD probability (Fig. 2). According to the MDC scale that we used, a final genetic diagnosis was achieved in over 90 % of patients with the highest MDC scores (5–8 points). In all such cases (with one exception for a neonate with *CPS1* mutation), variants were found exclusively in MD-related genes. The diagnostic yield was the lowest (36 %) in the patients with low MD suspicion (MDC score 2–3), and most of the variants in this group were present in non MD-related genes.

A similar correlation between detection rate and the level of MD probability was described recently in a similar patient group studied by WES at the Nijmegen Mitochondrial Centre [10]. However, our results differed from that study in terms of the scope of detected defects. In our cohort, mutations in *MTO1*, *TK2*, *C12orf65*, *COA6*, *TUFM*, *GFM1* were absent and the defects in nuclear encoded complex I subunits are different. This may be a result of random patient selection, but we should also take into account ethnic differences among European populations, e.g., the Slavonic vs. north-western European populations.

In addition, we identified six rare mtDNA pathogenic variants, not included in the common mutations screening i.e. m.9185T>C in *MTATP6* [33–35] and in mitochondrial DNA genes encoding complex I subunits, *MTND1* [36–38], *MTND3* and *MTND5* [39–42].

One-third (15/47) of the identified gene defects were discovered during last 10 years and relatively poorly characterized in terms of phenotype. These included *PGAP2* [43, 44], *ACAD9* [45, 46], *EARS2* [47], *SERAC1* [48], *SLC19A3* [49, 50], *MTFMT* [51], *SLC25A12* [52] as well as *VARS2* [53], *AIFM1* [54], *RARS2* [55], *RRM2B* [56], *PIGN* [44, 57], *ADCK3* [58, 59] which were described in just individual cases. Notably, most of these genes are generally absent from commercial NGS panels available at present.

It is worth emphasizing that in some cases WES allowed for a diagnosis *in statu nascendi*, that is, at the time of the first publication of the new gene. This concerned, for example, mutations in *CLPB* [25, 60], *PARS2* [26], *FBXL4* [61, 62] and recently added *TMEM126B* (data published on ESHG 2016 by Alston et al.), and *NAXE* [28] In one of the patients with the MD phenotype we identified potentially pathogenic variants in candidate *NDUFB8* which role in human pathology is under verification [Piekutowska-Abramczuk et al. submitted to SSIEM 2016].

According to published literature, every third paediatric MD case (approximately 30 % of all MD diagnoses in this age group) manifests clinically shortly after birth [12, 13]. The fatal outcome in such cases precludes transport to a reference centre and proper mitochondrial diagnostics. We have previously shown significantly reduced (up to ten times, about 3 % of all diagnoses) recognition of MD in this age group in Poland [16]. Therefore, neonates with suspected MD intentionally constituted a significant proportion of patients (47/113) undergoing WES in the present study.

Surprisingly, in the neonatal subgroup WES proved to be particularly useful, allowing identification of pathogenic variants in 24 various genes in 63.8 % of patients, including those without muscle biopsy or even autopsy. Our results extend the list recommended by Honzik [13] for neonatal MD diagnostics by at least 15 genes (MD-related: *RRM2B*, *CLPB*, *ACAD9*, *FBXL4*, *PC*, *AIFM1*, *SLC25A12*, *MTND5*, *NDUFS6* and non MD-related: *CPS1*, *PGAP2* and more).

In the LS subgroup WES expanded the set of patients from our centre diagnosed with complex I deficiency by three known genes: *NDUFS6* [63, 64], *NDUFV1* [65, 66], *NDUFS7* [67], a new candidate *NDUFB8* [68] and five *MTND*s mentioned above. Despite this, complex I deficiency continues to be underrepresented in our cohort in relation to complex IV deficiency because of the high carriage rate of *SURF1* mutations in Poland [69]. In a number of cases with basal ganglia brain changes, WES failed to show mutations in known LS-associated genes. This was especially the case in patients without lactic acidaemia and MDC scores below 5 (MD possible but not likely). We speculate that other, still unknown, genes or non-genetic factors might influence the occurrence of LS-brain changes.

Taken together, our results indicate that WES rather than targeted NGS should be the method of choice for MD testing, at least until all MD-associated genes are identified. Furthermore, the rationale for choosing WES in MD-suspected neonates is the non-specificity of symptoms and overlapping results of biochemical tests with non-mitochondrial errors of metabolism.

In 50.5 % the molecular variants were novel (Table 3). However, a number of recurrent rare pathogenic variants found in some recently discovered MD genes (p.Arg22* in *FBLX4*, p.Arg518Cys in *ACAD9*, p.Arg417* in *CLPB* and c.1822_1828+10delinsACCAACAGG in *SERAC1*) may extend the ethnic specificity of MD in the Polish population reported earlier by us for variants p.Glu140Lys in *SCO2* [14] and c.845_846delCT in *SURF1* genes [69]. Confirmation of these findings could facilitate in-house diagnostics in selected suspected cases.

## Conclusions

1. In a nationwide reference centre, WES provided positive results in >90 % of children with high likelihood of MD (MDC score above 4);
2. WES should be recommended for diagnostics of mitochondrial pathology considering remarkable representation of non MD-related genes among causal factors in patients with lower likelihood of MD, as well as a possibility to discover new mitochondrial genes;
3. WES significantly improves recognition of MD in newborns, even in the case of limited availability of appropriate diagnostic procedures;
4. Despite being a *sine qua non* for certain diagnoses 3-MGA is not a universal marker of mitochondrial dysfunction;
5. Recurrent variants recognized in some relatively new MD genes (*FBLX4*, *ACAD9*, and *CLPB*) may extend the known ethnic specificity of MD in the Polish population reported earlier for *SCO2* and *SURF1* variants.

## Additional file

10.1186/s12967-016-0930-9 Characteristics of 113 patients with probable/possible mitochondrial disease recruited for the study.

## Abbreviations

MD: mitochondrial disorders
WES: whole-exome sequencing
MDC: mitochondrial disease criteria
NGS: next generation sequencing
LA-uria: lactic aciduria
3-MGA-uria: 3-methylglutaconic aciduria
nDNA: nuclear DNA
