# Supplementary material for: New perspective in diagnostics of mitochondrial disorders: two years’ experience with whole-exome sequencing at a national paediatric centre
Source: J Transl Med. 2016 Jun 12;14:174. doi: 10.1186/s12967-016-0930-9 (PMC4903158; doi:10.1186/s12967-016-0930-9)
Supplement: Supplementary file 1 — 10.1186/s12967-016-0930-9 Characteristics of 113 patients with probable/possible mitochondrial disease recruited for the study. [file 12967_2016_930_MOESM1_ESM.docx]

**Table S1.** Characteristics of 113 patients with probable/possible mitochondrial disease recruited for the study.

| **ID patient** | **Age**  **of onset** | **Age**  **of death** | **Pregnancy**  **and perinatal history** | **Major symptoms and biochemical findings and family history** | **WES result** |
| --- | --- | --- | --- | --- | --- |
| 1 | 1 d | 6 d | PII DII; 40/3200/10 | Hypotony, decreased response to stimulus, severe LA, similar disease in brother | *NDUFS6* |
| 2 | fetal | 21 d | PI; 37/2830/7>8 | Severe liver failure, respiratory insufficiency, LA | *PRF1* |
| 3 | birth | Alive | PII DI; 35/2320/2>7 | Dysmorphy, psychomotor retardation, LA, muscle biopsy: RCC↓, ex post veryfication: mtDNA depletion | *FBXL4* |
| 4 | 5 y | Alive | PI; N/N/N | Steroid resistant nephrotic syndrome, migrene, deafness (high tones), LA, muscle biopsy: CI↓, CIV↓, high citric syntase activity | *not conclusive* |
| 5 | fetal | 6 mo | PI; 36/2400/3>6 | Severe LA, spasticity, neutropenia, 3-MGA (52-93 mmol/mol creat.) | *CLPB* |
| 6 | 2 mo | Alive | PI DI; N/4300/10 | EIEE, hypotony, dysmorphy, abnormal MRI, consanguinity | *PIGN* |
| 7 | 2 mo | Alive | PI (cs); N/2210/10 | Encephalopathy, seizures, hepatic failure, LS, LA, muscle biopsy: CI↓, ex post veification: specific MRI features | *EARS2* |
| 8 | 5y | Alive | PI DI; N/N/N | Mild CMP and 3-MGA revealed by study (of family at risk - sudden father's death, HCM at autopsy), during verification: 3-MGA excretion very low (traces) | *MYBPC3* |
| 9 | 8 mo | 25 mo | PI; 39/3160/9>10 | 3 mo: hypotony, sideroblastic anaemia (multiple blood transfusions), severe LA, progressive encephalopathy, muscle biopsy: CIV↓, increased iron level, mtDNA depletion | *COX10* |
| 10 | 3.5 y | Alive | PII; 39/3290/9 | LS, LA, muscle biopsy: CI↓ | *NDUFV1* |
| 11 | 7 y | Alive | PI; 40/3940/10 | Stroke-like episode, motor development retardation, mild LA, 3-MGA , during verification: 3-MGA excretion within reference range (5 mmol/mol creat.) | not conclusive |
| 12 | 8 mo | 24 mo | PI; N/N/N | Psychomotor retardation, myoclonic epilepsy, brain oedema, similar disease in brother | *APOA1BP* |
| 13 | 1 d | 11 d | PII DII; N/4190/10 | Cardio-respiratory failure, severe LA, 3-MGA (46 mmol/mol creat.), affected sister | *CPS1* |
| 14 | infancy | 7 y | PI; 37/2450/ND | Psychomotor retardation, FTT, CMP, hepatic cirrhosis, Ltx (2 y), muscle biopsy: unspecific changes, during verification: increased glycogen amount in liver, ↓brancher activity | *GBE1* |
| 15 | 2 d | Alive | PI; N/2950/9 | Hypotony, CMP, LA, 3-MGA (19 mmol/mol creat.), muscle biopsy: RRF, CI↓ | *ACAD9* |
| 16 | 3 mo | Alive | PI; 39/2880/10 | West syndrome, LS, 3-MGA, during verification: 3-MGA not increased (traces) | not conclusive |
| 17 | 12 mo | Alive | PIII DIII (cs); 40/4400/10 | Psychomotor retardation, slowly progressive piramidal-extrapiramidal syndrome, MRI: basal ganglia involvement, muscle biopsy: unspecific changes, similar disease in brother | not conclusive |
| 18 | 8 mo | Alive | PII DII; N/3400/10 | Dystonia, athetosis, MRI: LS-like (verification: bilateral striatal necrosis), muscle biopsy: normal range; affected brother | *ADAR* |
| 19 | 2 y | Alive | PII; N/N/N | Guillain-Barre episode, peripheral neuropathy, LA, increased pyruvate concentrations, low lactate/pyruvate ratio, muscle biopsy: unspecific changes, similar disease in brother | *PDHA1* |
| 20 | infancy | Alive | PI (cs); N/3840/7 | Deafness (4 mo), developmental regression (2.5 y), leukoencephalopathy | *PEX5* |
| 21 | 2 mo | 4 mo | PIV DIII; 37/2900/10 | Deafness, hypotony, neurological regression, marked LA, muscle biopsy: CI↓, CIV↓, during verigication: mtDNA depletion in muscle | *RRM2B* |
| 22 | 4 mo | Alive | PII DII (cs); 40/2450/10 | LS, CMP, FTT, cataract, LA, neutropenia, hyponatremia, 3-MGA (7-15 mmol/mol creat.), muscle biopsy: subsarcolemmal densities | *MTND1* |
| 23 | 1 mo | 3 mo | PII DII; 39/2440/10 | CMP, hypotony, LA, muscle biopsy: RRF, CI↓ | *ACAD9* |
| 24 | birth | Alive | PII DII (cs); N/2500/4 | Muscle hypotony, ptosis, epilepsy (8 mo), LA, muscle biopsy: unspecific changes | *SLC25A12* |
| 25 | birth | 32 mo | PI (twin); ND/ND/ND | Encephalopathy, CMP, respiratory failure, LA, muscle biopsy: SMA-like pattern, CI↓, CIV↓ | *AIFM1* |
| 26 | 1 d | Alive | PI; 38/2350/9 | Psychomotor retardation, LS, leukodystrophy, LA, muscle biopsy CI↓ | *NDUFB8* |
| 27 | birth | 2 mo | PII DII; N/N/5>8 | Severe LA, spasticity, neutropenia, 3-MGA (133-398 mmol/mol creat.), similar disease in brother | *CLPB* |
| 28 | 1 d | 12 mo | PII; 38/1900/8/9 | DCM, recurrent cardiac insufficiency, 3-MGA excretion consistently within normal range, found increased only once just before death (40-50 mmol/mol creat.), muscle biopsy: RCC↓ | *TAZ* |
| 29 | birth | Alive | PI (cs); N/3650/9 | Progressive encephalopathy, stridor, LA, increase excretion of lactate, pyruvate and ketone bodies, low lactate/pyruvate ratio, similarly affected brother, muscle biopsy: "lipid storage myopathy" | *PC* |
| 30 | birth | 3 y | PIII DIII (cs); N/4650/8 | Hypotony, seizures, muscular dystrophy suspicion, similar disease in brother, very long chain fatty acids concentration not measured, muscle biopsy: unspecific changes | *HSD17B4* |
| 31 | 12 mo | Alive | PIII DII; N/N/N | LS, slow disease progression, muscle biopsy: unspecific changes | *DLD* |
| 32 | 9 y | 9 y | PII; N/N/N | Ptosis, sudden neurological regression, typical LS chancges on brain autopsy | *MTATP6* |
| 33 | 2 y | Alive | PIII; N/N/N | Familiar blindness, epilepsy, stroke (7 y); similar disease in mother and sister | *OPA1* |
| 34 | 3 y | Alive | PII DII; ND/ND/ND | Arterial hypertension, CMP, LS, LA, muscle biopsy: CI↓, CIV↓ | *MTND5* |
| 35 | 2 mo | 10 mo | PI; N/3720/10 | Progressive encephalomyopathy, HCM, LA, muscle biopsy: "lipid storage myopathy" | *MTND5* |
| 36 | 20 mo | 24 mo | PIV DIII; N/3200/10 | LS, macrocytic anemia, LA, muscle biopsy: CIV↓ | *COX10* |
| 37 | birth | Alive | PI; 34/1530/9 | Liver dysfunction, FTT, neurological regression, LS, 3-MGA (22-30 mmol/mol creat.), muscle biopsy: at normal range | *SERAC1* |
| 38 | 15 y | Alive | PII (twin); N/2500/10 | CMP, heart transplantation, muscle biopsy: unspecific changes, similar disease in a number of family members | *DMD* |
| 39 | 5 y | Alive | PI; 37/1650/ 8 | Stroke-like episodes, muscle biopsy: unspecific changes; affected many relatives | *CACNA1A* |
| 40 | 1 d | 10 d | PII; N/3300/9 | increased NH_3_↑, LA, 3-MGA (88 mmol/mol creat.); affected brother | *CPS1* |
| 41 | 3 mo | Alive | PI; 41/3780/ 10 | West syndrome, developmental regression, cerebellar atrophy, muscle biopsy: unspecific changes; affected brother | *RARS2* |
| 42 | 3 mo | Alive | PIII; 40/3240/10 | EIEE | *GFAP* |
| 43 | 46 y | 47 y | ND; N/N/N | Cerebral haemorrhagy, respiratory failure, death, suspicion of SMA-like disorder | not conclusive |
| 44 | 46 y | Alive | ND; N/N/N | Slowly progressive neuromuscular disorder, affected many relatives in three generations | not conclusive |
| 45 | 3 y | Alive | PVI DVI; N/3600/10 | Epilepsy, increased creatine phosphokinase concentration, muscle dystrophy features | *DYSF* |
| 46 | 3 mo | Alive | PI; N/N/N | Developmental delay, general muscle hypotony, CMP suspicion, muscle biopsy: unspecific changes | not conclusive |
| 47 | 4 mo | Alive | PI; 41/360/7>10 | EIEE, hypsarythmia, refux | *SCN2A* |
| 48 | 2 y | Alive | PII; 37/2660/5>8 | Stunded growth, syncope episodes, VPA intolerance | not conclusive |
| 49 | birth | 6 mo | PIV; 31/1950/9 | Prematurity, CMP, hypoplastic kidney, renal hyperkalemic acidosis, LA ; similar disease in sibs | not conclusive |
| 50 | fetal | 3 d | PIII DIII (ve); 38/3600/5 | Congenital heart defect, mtDNA depletion in liver | not conclusive |
| 51 | 2 wk | 2 y | PI DI; N/3880/10 | Encephalopathy, cataract, CMP, lactate, branched chain aminoacids, muscle biopsy: mtDNA depletion | *RRM2B* |
| 52 | birth | Alive | PII DI; N/3700/ 9 | Psychomotor retardation, dysmorphy (Cornelia de Lange phenotype), LA | *FBXL4* |
| 53 | early infancy | Alive | PI DI; 40/2875/N | Muscle hypotony, proximal tubulopathy, LA, muscle biopsy: single RRF, CI↓ | *ACAD9* |
| 54 | 8 mo | 21 mo | PIII DIII; N/3600/10 | Progressive encephalopathy, truncal hypotony, spasticity, neutropenia, LA, MRI: leukodystrophy, muscle biopsy: SMA-like pattern, histochemistry not done; similar disease in sister | *SCO2* |
| 55 | 13 y | Alive | PII DII; 40/3300/10 | Psychomotor retardation, tremor, uncertain gait, LA, muscle biopsy: unspecific changes | *FBXL4* |
| 56 | 3 mo | Alive | PII DII (cs); 41/3130/10 | Psychomotor retardation, 3 mo: West syndrome, brain atrophy, LA | *PDHA1* |
| 57 | 14 mo | Alive | PII DII; 40/310/10 | Psychomotor retardation, ptosis, LS, LA, ketosis, muscle biopsy: CI↓ | *MTND3* |
| 58 | 12 mo | Alive | PIII DI; 40/4350/10 | Psychomotor retardation, LS, LA, ketosis, muscle biopsy: CI↓ | *SLC19A3* |
| 59 | 1 d | Alive | PII DII; 37/2150/10 | CMP, hypoplastic kidney, hyperkalemic acidosis, LA, muscle biopsy:CI↓ | *TMEM126B* |
| 60 | birth | 8.5 y | PII DII; 38/3150/9 | Congenital microcephaly, EIEE, LS-like, DCM, renal insufficiency, muscle biopsy: at normal range; affected sibs | *PARS2* |
| 61 | 2.5 y | 26 y | PII DII; N/3600/10 | Hypertransaminasaemia, myopathy, myoclonic epilepsy, CMP, LA, muscle biopsy: RRF, RCC↓ | *ADCK3* |
| 62 | 2 wk | 4.5 mo | PII DII (forceps) N/4100/10 | Progressive hepatic cirrhosis, CMP, renal dysembrioplasia, LA, muscle biopsy: CI↓; similar disease in brother | not conclusive |
| 63 | 8 y | Alive | ND; N/N/N | Myoclonic epilepsy, progressive extrapiramidal syndrome, muscle biopsy: technical difficulties; child adopted | not conclusive |
| 64 | 5 wk | 7 mo | PIII DIII (cs); 38/3040/10 | Hypotonic respiratory failure, progressive encephalopathy, LS, muscle biopsy: unspecific changes, CI↓ | *MTND1* |
| 65 | 5 d | Alive | PI; 39/3590/9>10 | EIEE, 8 mo: West syndrome, LS, muscle biopsy: at normal range | *CDKL5* |
| 66 | birth | Alive | PII DII; N/2900/10 | Microcephaly (31 cm at birth), congenital encephalomyopathy, LA, muscle biopsy: "lipid storage myopathy", RCC activity in normal range | *PDHA1* |
| 67 | fetal | Alive | PIV DII; N/N/7 | Spasticity at birth, LA, hypoglycemia, transient neutropenia, 3-MGA (92-98 mmol/mol creat.) | *CLPB* |
| 68 | infancy | Alive | PI DI; 41/3400/N | Progressive hypotony, encephalopathy, LA, MRI: basal ganglia involvement, muscle biopsy: at normal range | *PDHA1* |
| 69 | 8 mo | Alive | ND | EIEE, hypsarytmia, MMA (maternal vit B12 deficit) | not conclusive |
| 70 | 1.5 mo | 8.5 mo | PIII DIII; 41/3850/10 | Recurrent episodes of severe LA with ketosis, hypotony, retardation of motor development, hyperglycemia, muscle biopsy: SMA-like pattern, CIV↓ | *EARS2* |
| 71 | 1-2 d | 3 mo | PI; 40/3100/10 | Dysmorphy, truncal hypotony, opistotonus, hypotrophy, cholestasis, LA, muscle biopsy: "lipid storage myopathy", RCC↓ | *PC* |
| 72 | birth | 4 mo | PII DII; 33/2200/6 | Falot syndrome, acute liver failure, COX deficiency in muscle, mtDNA depletion | not conclusive |
| 73 | birth | Alive | PI; 39/2940/8 | Psychomotor retardation, generalized weakness, seizures; muscle biopsy: unspecific changes, ex post verification: sustained hyperphosphatasaemia (neglected) | *PGAP2* |
| 74 | 14 y | Alive | PII DII; N/3350/10 | Migrene, extrapiramidal symptoms provoked by exercise, marfanoid phenotype, muscle biopsy: CI↓ | not conclusive |
| 75 | 5 d | 5 mo | PIII DIII (cs); 36/ 2150/8 | Haemorrhagic diathesis, hepatic and renal failure, multifocal hyperechogenisity, muscle biopsy: CI↓; similar disease in brother | *NDUFS7* |
| 76 | 4 y | Alive | PI DI; N/3500/10 | Cerebral palsy, strabismus, extrapyramidal syndrome, LS, muscle biopsy: at normal range | not conclusive |
| 77 | 1 d | 20 mo | PII DII; 40/3060/6 | Hypotony, respiratory failure, seizures, optic atrophy, cerebral atrophy, LS-like, muscle biopsy: unspecific changes | not conclusive |
| 78 | 14 y | Alive | PIII DII; ND/ND/ND | Progressive "brady-tachy" syndrome, dementia (autistic behavior since 3 y), 3-MGA traces | not conclusive |
| 79 | 5 y | Alive | PI; 36/3250/9 | Neurological regression, spastic diplegia (psychomotor retardation in infancy), muscle biopsy: CI↓ | not conclusive |
| 80 | 18 mo | 18 mo | PI; 39/3600/10 | Hemolytic-uremic syndrome, 3-MGA (26-65 mmol/mol creat in sister.) | not conclusive |
| 81 | 1 d | 4 y | PV DV (cs); N/2900/10 | Trembling, hypoglycemia, LA, recurrent seizures (similar disease of four sibs), muscle biopsy: CIV↓ | not conclusive |
| 82 | 3 mo | Alive | PII DII; N/3390/9 | Psychomotor retardation, hypotrophy, in 13 mo: LS | not conclusive |
| 83 | 1 d | unknown | PI; 34/2300/7 | Respiratory failure, floppiness, EMG: myogenic features, ECG: repolarization abnormalities, muscle biopsy: muscle atrophy; OXPHOS technical difficulties | not conclusive |
| 84 | 1 wk | Alive | PI (cs); 36/2400/10 | Icterus, hypoglycemia, hemorthagic diathesis, progressive liver failure, mild LA, Ltx (3 mo), muscle biopsy: fatty accumulation, CIV↓ | not conclusive |
| 85 | 2 mo | 6 mo | PII DII; N/N/N | Spasticity, myoclonic episods, psychomotor retardation, LS | not conclusive |
| 86 | 2 mo | 2 y | PI DI; N/3320/10 | EIEE | not conclusive |
| 87 | 3 mo | Alive | ND; N/N/N | EIEE, child adopted, consanguinity; similar disease in sibs | not conclusive |
| 88 | 3 y | Alive | PIII DII; N/N/N | Progressive blindness, psychomotor retardation, ex post verification: ceroidlipofuscin accumulation in conjunctiva; affected sister | *CLN3* |
| 89 | birth | unknown | PII DI (cs); 40/3000/5 | Hypotony, respiratory failure, CMP, LS, muscle biopsy: CFTD (congenital fiber type disproportion) , RCC↓ | not conclusive |
| 90 | 2 mo | 21 mo | PI DI; N/2730/10 | Psychomotor regress, CMP, leukodystrophy retinitis pigmentosa, renal failure, LA, hyperglycemia, muscle biopsy: CI↓ | not conclusive |
| 91 | 2 mo | 2.5 mo | PII DI (cs); N/4670/10 | Cholestasis, progressive liver failure, CMP, LA, muscle biopsy: technical difficulties | *MTFMT* |
| 92 | 14 y | Alive | PI; N/N/N | Progressive liver cirrhosis (nodular regenerative hyperplasia), 3-MGA (5-4 mmol/mol creat.) | not conclusive |
| 93 | birth | 14 d | PI; 41/3100/10 | 3 day: infection, multiorgan insufficiency, liver necrosis, mtDNA depletion in liver | not conclusive |
| 94 | birth | Alive | PV (cs, twin 2); 31/1760/4>7 | Psychomotor retardation, deafness, sudden cardiac deaths of two sibs, 3-MGA (13 mmol/mol creat.); consanguinity; miscarriage | not conclusive |
| 95 | birth | Alive | PII DI; 40/3000/10 | Multiorgan involvement (liver, kidney, hemopoethic system), skeletal dysplasia, growth failure, muscle biopsy: CI↓ | *SBDS* |
| 96 | 4 mo | 2 y | PI; N/N/N | Acute respiratory failure, cardiac arrest, brain oedema, drug resistant epilepsy, brain atrophy, infections | not conclusive |
| 97 | 1 d | ND | PIII DIII; 40/3420/10 | Stridor, CMP, severe LA, muscle biopsy: at normal range; similar disease in sibs | *VARS2* |
| 98 | 2 d | 8 mo | PII (cs); 41/4250/10 | restrictive CMP, pulmonary hypertension, 3-MGA (4-33 mmol/mol creat.) | not conclusive |
| 99 | 18 y | Alive | ND; N/N/N | Progressive ataxia | not conclusive |
| 100 | 6 mo | 6 mo | PII DI; 40/2880/9 | Acute liver failure, brain oedema, LA, death within 9 days, muscle biopsy: unspecific changes | not conclusive |
| 101 | 8 mo | Alive | ND | EIEE, LS | not conclusive |
| 102 | 22 mo | Alive | PII DII; N/N/N | Hypotony, psychomotor retardation, LS | not conclusive |
| 103 | birth | 18 mo | PIV DIV; 36/2180/ND | DCM, 3-MGA traces | not conclusive |
| 104 | 5.5 y | Alive | PII DII; N/2900/10 | Mild choreoatetosis, basal ganglia involvement, muscle biopsy: unspecific changes, affected persons in three generations | not conclusive |
| 105 | 1 mo | 3 mo | ND | Severe LA | not conclusive |
| 106 | birth | Alive | PI (cs); 40/ND/6>9 | Floppy child on respirator, epilepsy, optic atrophy, brain atrophy, VPA intolerance, muscle biopsy: unspecific changes | *MECP2* |
| 107 | 3 mo | Alive | PIII DIII; 41/3000/10 | Familial progressive leukoencephalopathy (two boys), brain atrophy, prelingual hearing loss, cataract, muscle biopsy: unspecific changes | not conclusive |
| 108 | birth | Alive | PI; 35/2100/ND | Floppy child, insufficient respiration, protracted artificial ventilation, cerebellar vermis atrophy, muscle biopsy: unspecific changes | not conclusive |
| 109 | birth | 8 mo | PIII DIII; N/4050/8 | EIEE, progressive brain atrophy, similar disease in brother | *SLC19A3* |
| 110 | 6 mo | Alive | ND; N/N/N | Progressive EIEE, impaired myelinization, brain atrophy, muscle biopsy: technical difficulties | not conclusive |
| 111 | birth | 6 mo | PII DII (cs); 39/2500/3>7 | Spasticity at birth, convulsions, apnoe, progressive CMP | not conclusive |
| 112 | fetal | 6 d | PI (cs); 38/3330/2 | Congenital atrioventricular block, multioorgan failure, mtDNA depletion found at autopsy | not conclusive |
| 113 | 9 mo | 24 mo | PI; 41/N/10 | 8 mo: drug-resistent seizures; neurological regression; muscle biopsy: at normal range | *POLG* |

Abbreviations: d, day; mo, month; y, year; wk, week; N, reference range without precise data; P, pregnancy; D, delivery; hbd, weeks of gestation; cs, caesarean section; ve, vacuum extractor; ND, no data; LA, plasma lactate concentration <20 mg/dl; 3-MGA, 3-methylglutaconic acid urinary excretion >20 mmol/mol creatinine; RCC↓, multiple respiratory chain complex deficiency; CI↓, complex I deficiency; CIV↓ , complex IV deficiency; LS, Leigh syndrome; EIEE, early infantile epileptic encephalopathy; MRI, magnetic resonance imaging; CMP, cardiomyopathy; DCM, dilated cardiomyopathy; HCM, hypertrophic cardiomyopathy; FTT, failure to thrive; RRF, ragged-red fibers; Ltx, liver transplantation; VPA, valproic acid; mtDNA depletion, mtDNA/nDNA ratio >30*%* of reference group (n=43); muscle biopsy: at normal range, complexes I-IV and citric synthase activities within laboratory reference.
